# Performance in Kahoot! activities as predictive of exam performance

**DOI:** 10.1186/s12909-023-04379-x

**Published:** 2023-06-06

**Authors:** MC Garza, S Olivan, E Monleón, Ana Isabel Cisneros, A García-Barrios, I Ochoa, J Whyte, I Lamiquiz-Moneo

**Affiliations:** 1 Department of Human Anatomy and Histology, School Medicine, University of Zaragoza, Zaragoza, Spain; 2 Tissue Microenvironment (TME) Lab. Aragón Institute of Engineering Research (I3A), University of Zaragoza, Zaragoza, Spain; 3 Institute for Health Research Aragón (IIS Aragón), Zaragoza, Spain; 4 Biomedical Research Networking Center in Bioengineering, Biomaterials, and Nanomedicine, CIBER- BBN, Planta, Spain; 5 Medical and Genetic Research Group (GIIS099) IIS Aragón, Zaragoza, Spain; 6 Unidad de Lípidos, IIS Aragón, CIBERCV, Hospital Universitario Miguel Servet, Avda. Isabel La Católica 1-3, Zaragoza, 50009 Spain

**Keywords:** Game-based learning, Kahoot!, Predictive tool, Neuroanatomy, Human histology, Medicine

## Abstract

**Background:**

Game-based learning (GBL) is effective for increasing participation, creativity, and student motivation. However, the discriminative value of GBL for knowledge acquisition has not yet been proven. The aim of this study is to assess the value of Kahoot! as a discriminative tool for formative assessment in medical education in two different subjects.

**Methods:**

A prospective experimental study was conducted on a sample of 173 students enrolled in neuroanatomy (2021–2022). One hundred twenty-five students individually completed the Kahoot! prior to the final exam. In addition, students enrolled in human histology during two academic courses were included in the study. The control group course (2018–2019) received a traditional teaching methodology (N = 211), while Kahoot! was implemented during 2020–2021 (N = 200). All students completed similar final exams for neuroanatomy and human histology based on theory tests and image exams.

**Results:**

The correlation between the Kahoot score and the final grade was analyzed for all students enrolled in neuroanatomy who completed both exercises. The correlation between the Kahoot exercise and the theory test, image exam and final grade was significantly positive in all cases (r = 0.334 p < 0.001, r = 0.278 p = 0.002 and r = 0.355 p < 0.001, respectively). Moreover, students who completed the Kahoot! exercise obtained significantly higher grades in all parts of the exam. Regarding human histology, the theory tests, image exams and final grades were significantly higher when using Kahoot! versus the “traditional” methodology (p < 0.001, p < 0.001 and p = 0.014, respectively).

**Conclusions:**

Our study demonstrates for the first time that Kahoot! can be used to improve and predict the final grade in medical education subjects.

**Supplementary Information:**

The online version contains supplementary material available at 10.1186/s12909-023-04379-x.

## Introduction

A good anatomical and histological knowledge base is decisive for the career development of physicians of any specialty. These areas are considered “basic subjects” within the medical field and concepts are rarely updated, establishing a false sense of stagnation. As a result, these subjects may be characterized by long-standing traditional methods of teaching and assessment. Recently, two factors have revolutionized the teaching methodology of these basic subjects: the need to adapt teaching methodologies to “Generation Z” and the need to reduce face-to-face class learning due to the COVID-19 pandemic [1–7].

Generation Z, young people born between 1997 and 2012, is the first digital generation; they have had lifelong access to new virtual tools and other technological applications through electronic devices (tablets, mobile phones or laptops), which gives them unprecedented technological skills [8–10]. This “cultural and generational” change in students is supported by the Higher Education Space (EESS), which embraces a commitment to methodological renewal that improves the quality of teaching and encourages students’ participation and motivation in higher education classrooms [11, 12]. Game-based learning (GBL) appears to be an alternative teaching methodology to improve the teaching-learning process, as suggested by the EESS [13]. The numerous benefits of GBL in terms of fostering student participation and creativity and improving students’ motivation when facing different subjects have been widely described [14–17]. Furthermore, the possibility of using GBL remotely is an advantage that accelerated the establishment of new methodologies when face-to-face classes on campus were restricted by the COVID-19 pandemic [18].

One of the widely used online game-based platforms is Kahoot!, which has been adopted in various degree programs and universities [19] and has resulted in learning gains by improving students’ performance and engagement [20]. Several studies have explored students’ utilization, outcome scores, and perceptions of learning with the application of Kahoot! in histology, anatomy, and medical education [21]. In medical education, the implementation of Kahoot! has been shown to motivate students to study, assist them in developing self-directed learning, and help them focus on important concepts [22, 23]. In addition, Kahoot! has been proposed as a promising tool for facilitating formative assessment [24]. However, to our knowledge, most studies have addressed this concept from a qualitative perspective. Studies have not evaluated whether GBL tools, specifically the Kahoot! platform, can allow the students or the concepts with the greatest deficiencies to be identified and reinforced. Therefore, the aim of this study was to assess the value of Kahoot! as a predictive tool for formative evaluation in medical education. We examined this tool in two different subjects, neuroanatomy and histology, to consider whether these teaching methodologies are transferable to other subjects and areas of knowledge.

## Materials and methods

### Study design

#### Neuroanatomical group

A prospective experimental study was conducted on a sample of 125 students belonging to two different class groups. They were 173 s-year students enrolled in neuroanatomy in the degree in medicine at the University of Zaragoza (Spain) during the 2021–2022 academic year (Supplementary Table 1). This subject is taught during the second year of the degree and is equivalent to 9 credits of the European Credit Transfer and Accumulation System (ECTS). The teaching method is based on master classes together with the development of practices in the dissection room, with cadavers, plan anatomy atlases, dissected brain sections stained with the Weigert method, anatomy mockups and natural pieces. There are 4–5 theoretical classes and 2 h of practice per student per week (Supplementary Table 2). At the end of the theoretical content prior to the final exam, the GBL tool Kahoot! was used to reinforce and evaluate the students individually with the subsequent resolution of the questions in class. The students completed the Kahoot! exercise in class individually using a personal number assigned for their identification with the aim of treating the data anonymously. The same personal number was used for the students’ identification on the final exam. The final exam was conducted two weeks after the use of Kahoot! in the classroom. This exam consisted of two parts: (a) a theory exam with 30 multiple-choice questions, which represented 60% of the final grade, and (b) an image exam with different image questions about cadavers, anatomy atlases and natural pieces, which represented 30% of the final grade. The remaining 10% of the final grade was obtained from continuous evaluation, attendance practices, seminars, and the preparation of clinical cases related to neuroanatomy.

#### Human histology group

To analyze Kahoot’s effectiveness in improving students’ grades, the data from the final exam of 2 distinct academic years of human histology were compared: The 2020–2021 course (n = 200) in which Kahoot! was implemented and the 2018–2019 course (n = 211) with a “traditional” teaching/learning methodology (Supplementary Table 1). Human histology is taught during the second year of the degree and is equivalent to 6 ECTS credits. There are 3 theoretical classes and 2 h of practice per student per week. The teaching method is based on master classes together with the development of practices and seminars in the laboratory room with the use of a microscope. Kahoot! seminars were held throughout the 2020–2021 academic year at the end of each seminar to reinforce important concepts followed by identifying wrong answers. In 2018–2019, a traditional teaching/learning methodology image review was performed after each seminar. The final exam was similar to neuroanatomy with two parts: (a) theory test with 50 multiple-choice questions (50% of the final grade) and (b) an image exam with 5 micrographs of organs (50% of the final grade). In addition, there was a practical exam that consisted of recognizing and describing 5 histological sections under the microscope and describing all the structures the student recognized. The final grade was the average of the final exam and the practical exam. In both courses, 2018–2019 and 2020–2021, the course was consistent with regard to the teaching staff and the curriculum content in human histology, ensuring an identical educational experience.

### Game-based learning tool

The GBL tool Kahoot! was implemented during the 2021–2022 academic year in the neuroanatomy group and in 2020–2021 in the histology subject as an innovative teaching method with the aim of improving previously acquired skills and competences as well as conducting continuous assessment and evaluation of the prediction value of this method. Considering the content that had to be evaluated in the courses, 10 multiple-choice questions were designed in neuroanatomy and 25 in histology with only one possible correct answer with a limited response time of 20 s. The 10 questions evaluated with Kahoot! in neuroanatomy are included in Supplementary Tables 3 and correspond to the theoretical content taught in the subject. The questions included in this Kahoot! were made by Associate Professor ILM and reviewed by the professors responsible for the subject, JW and AIC. They contained questions on all the topics studied. In the case of histology, each question was based on a specific microscope image corresponding to the topics studied. Before the students began using the educational game, they were given instructions and the possibility of either downloading the application on their mobile devices or using it online. At the end of the last theoretical class in neuroanatomy, the students were invited to perform a Kahoot! that had been prepared with the aim of assessing their level of knowledge and skills by both the student and the teacher. The 10 questions evaluated with Kahoot! in neuroanatomy are included in Supplementary Tables 3 and correspond to the theoretical content of the subject. In the case of the histology subject, a total of 10 Kahoot! were performed during the year, corresponding to each of the topics studied with an average of 25 questions per Kahoot!. An example of one Kahoot! performed in histology is included in Supplementary Table 4. The human histology Kahoot! was made by Associate Professor MCG and reviewed by the professors responsible for the subject, EM, SO and IO. It contained questions on all the topics studied.

### Statistical analysis

Normal distribution of all continuous variables was analyzed by the Shapiro‒Wilk test and histogram distribution prior to the statistical analysis. Continuous variables were expressed as the mean ± SD or median (25th percentile − 75th percentile) as applicable, and categorical (nominal) variables were reported as percentages of the total sample. Differences between independent variables were calculated by Student’s t test, the Mann‒Whitney U test or the Wilcoxon test, as appropriate, while categorical variables were compared using the chi-squared test. Correlation between the Kahoot! exam and different parts of the final exam were analyzed by the Spearman method for all students who completed the Kahoot exercise and exams in neuroanatomy. The positive predictive value was calculated by a score of passing, notable and outstanding between the Kahoot! exercise and the final exam. Regression analyses were conducted to identify the final grade percentage that could be explained by the grade obtained in the Kahoot! exercise using the theory, image and final grade as dependent variables and the Kahoot! score as an independent variable. Low-end outliers were identified as random responders and excluded from the final analysis [25]. All statistical analyses were performed with R version 3.5.0, and significance was set at p < 0.05.

## Results

Neuroanatomy and human histology are subjects studied in the second semester of the second year of medical school. Both subjects are mandatory in medical school for students 19–20 years old. In human histology, there was a predominance of women, with 159 (75.0%) compared to only 53 men (25.0%) in the 2018–2019 academic year and 138 (69.3%) women and 61 (30.6%) men in 2020–2021. A similar sex distribution was observed in neuroanatomy, with 48 (27.4%) men and 127 (72.6%) women enrolled in the academic year 2021–2022. The percentage of students enrolled in the course for the first time was slightly higher in neuroanatomy, with 96.5% versus 87% for human histology (Supplementary Table 1).

Table 1 shows each of the 10 questions analyzed, the percentage of correct answers, the average response time and the comparison between the response time of those who answered correctly or incorrectly. An average of 67.3% students answered correctly, and in most cases, the response time was significantly shorter among the students who answered correctly than among those who answered incorrectly. However, it is interesting to highlight that questions with more than 90% correct answers or more than 60% incorrect answers did not show a significant difference between the time to answer among students who answered correctly and those who answered incorrectly (*p* = 0.202 and *p* = 0.488, respectively).

**Table 1:** Analysis of content-related responses with Kahoot! exercise in the Neuroanatomy group

	Correct answer, n (%)	Average response time (sec)	Average response time in those who get the answer right (sec)	Average response time on those that fail to respond (sec)	*p*	Item difficulty index^1^	Item discrimination^2^
**Question 1**	74 (59.2%)	9.42 ± 5.03	7.99 ± 4.92	11.4 ± 4.56	< 0.001	0.592	0.650
**Question 2**	84 (67.2%)	11.7 ± 4.95	10.63 ± 4.43	13.9 ± 4.30	0.001	0.672	0.700
**Question 3**	79 (63.2%)	11.8 ± 4.54	10.75 ± 4.65	13.7 ± 3.85	< 0.001	0.632	0.300
**Question 4**:	77 (61.6%)	13.04 ± 4.89	12.1 ± 4.89	14.48 ± 4.64	0.005	0.616	0.450
**Question 5**	50 (40.0%)	7.74 ± 5.22	7.02 ± 4.81	8.27 ± 5.48	0.202	0.400	0.650
**Question 6**	117 (93.6%)	10.60 ± 3.88	10.52 ± 3.66	11.7 ± 5.84	0.488	0.936	0.650
**Question 7**	79 (63.2%)	11.6 ± 5.37	10.12 ± 5.33	13.9 ± 4.67	< 0.001	0.632	0.250
**Question 8**	70 (56.0%)	8.21 ± 5.68	6.57 ± 4.69	10.29 ± 6.17	< 0.001	0.560	0.300
**Question 9**	97 (77.6%)	9.96 ± 5.00	8.99 ± 4.62	13.10 ± 5.01	< 0.001	0.776	0.200
**Question 10**	111 (88.8%)	5.42 ± 5.17	4.57 ± 3.11	10.89 ± 10.23	0.012	0.888	0.650

Quantitative variables were expressed as mean ± standard deviation, while qualitative variables were expressed as n (%). The p value was calculated by Chi-Squared test. ^1^Item difficulty index was calculated dividing the number of correct responses between the total number of responses (which includes both correct and incorrect responses)

^2^Item discrimination was calculated following the next formula D =(UG-LG)/n, when UG is the number of correct answers of students in the upper 27%, LG is the number of correct answers of students in the lower 27% and n is the total of response

**Fig. 1:**
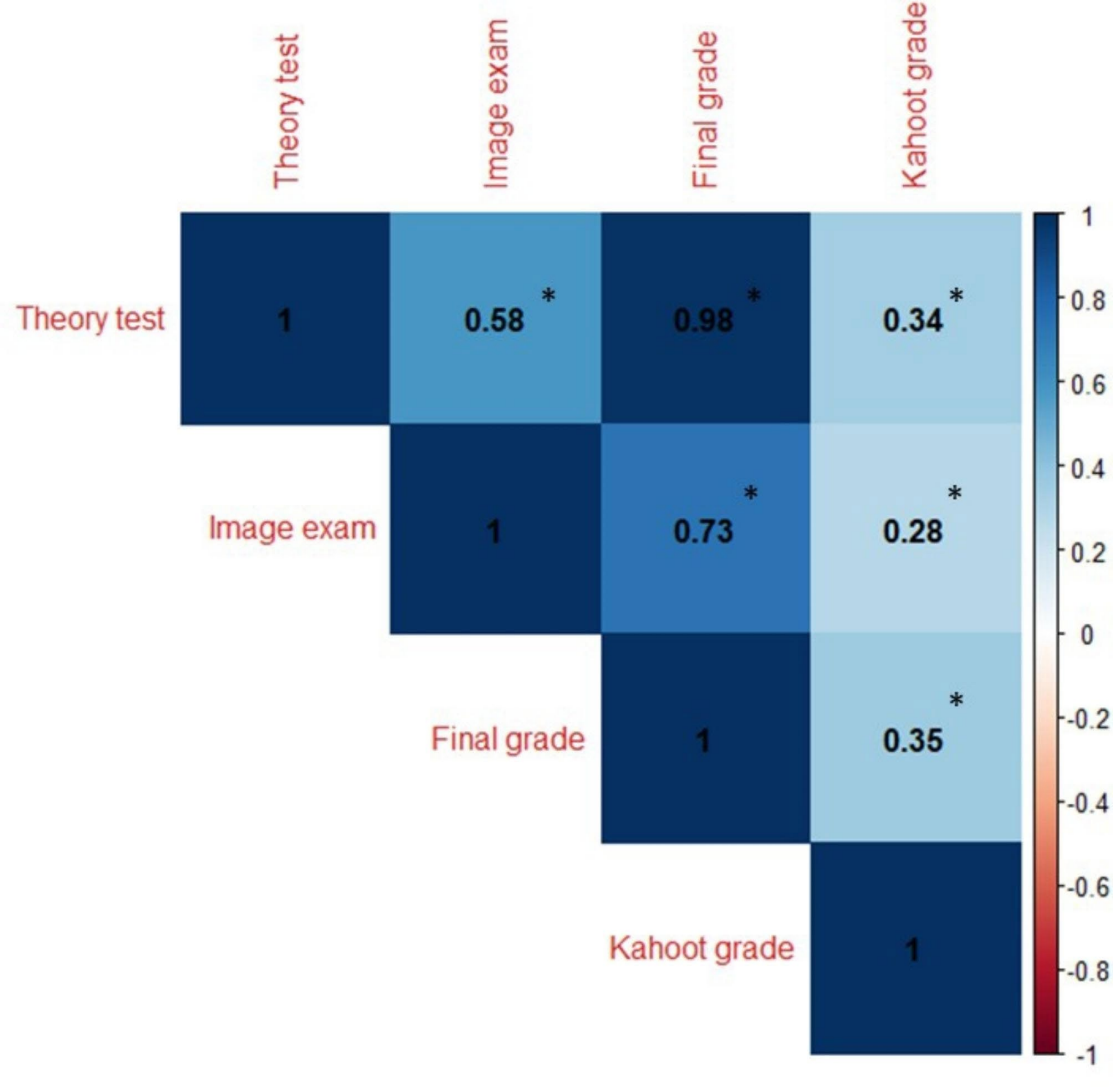
Correlation between the Kahoot! exam and different parts of the final exam. *Denotes p < 0.05 calculated by the Spearman method

When we analyzed whether a relationship existed between Kahoot! grade and the final exam, we found that the Kahoot! exercise showed a significant positive correlation with the theory test, image exam and final grade (r = 0.334 with *p* < 0.001, r = 0.278 with *p* = 0.002 and r = 0.355 with *p* < 0.001, respectively, Fig. 1). The theory test grade showed a positive significant correlation with the image exam (r = 0.585 with *p* < 0.001, Fig. 1). In addition, the regression analysis showed that the Kahoot! grade explained up to 6.7% of the final exam grade (p = 0.002) and up to 4.3% of the theory grade (p = 0.011) but did not seem to have a significant association with the imaging exam (p = 0.174).

Table 2 shows the number of students who obtained passing, notable or outstanding grades in the Kahoot! exercise and final exam. The positive predictive values reported were 60.3%, 96.0% and 26.5% for passing, notable and outstanding grades, respectively.

**Table 2:** Number of students who obtained passing, notable or outstanding in Kahoot! exercise and final exam

	Number of subjects who obtained these marks in the Kahoot exercise	Number of subjects who obtained these marks in the final exam
Passing (5 or more points)	73	121
Notable (marks from 7 to 8.9 points)	48	50
Outstanding (9 or more points)	18	68

Table 3 shows the comparison between the overall exam between students who completed the Kahoot! exercise versus students who did not complete the Kahoot! exercise. The data show that students who completed the Kahoot! exercise had significantly higher grades on the theory exam, image exam and overall grade than students who did not complete the Kahoot! exercise (*p* < 0.001, in all cases, Table 3).

**Table 3:** Comparison between in the overall exam between students enrolled in Neuroanatomy subject 2021-22, who completed the Kahoot! exercise versus students who did not complete the Kahoot! exercise

	Students who completed the Kahoot exercise (N = 125)	Students who did not complete Kahoot exercise (N = 48)	*p*
Final grade	8.84 ± 0.94	7.93 ± 1.34	< 0.001
Image exam	9.07 ± 1.25	6.98 ± 3.48	< 0.001
Theory exam	8.43 ± 1.42	6.51 ± 2.83	< 0.001

Quantitative variables were expressed as mean ± standard deviation. The p value was calculated by Wilcoxon test

Finally, to analyze whether Kahoot! could be a good tool to assess knowledge access in other subjects, we evaluated the differences between the theory exams, practical tests and image exams in two different years, one with the use of Kahoot! after each seminar and the other with the traditional teaching model in human histology. Table 4 shows that both the theoretical exam and the image exam as well as the global grade were significantly higher in the year in which the Kahoot! tool was used after each seminar than the year in which the class was taught in a traditional way (*p* < 0.001, *p* < 0.001 and *p* = 0.014, respectively).

**Table 4:** Comparison in the histology final mark between years in which the Kahoot! methodology was used versus years in which the Kahoot! methodology was not used

	Year in which the Kahoot methodology was used (N = 200)	Year in which the Kahoot methodology did not used (N = 211)	*p*
Final grade	7.02 ± 1.94	6.81 ± 1.50	0.014
Practice exam	6.13 ± 2.93	6.97 ± 1.75	0.131
Theory exam	6.18 ± 2.98	5.37 ± 2.76	< 0.001
Image exam	6.68 ± 3.12	6.03 ± 2.94	< 0.001

Quantitative variables were expressed as mean ± standard deviation. The p value was calculated by U Mann Whitney test

### Discussion

Face-to-face teaching does not improve student grades, but it has been demonstrated to influence students’ knowledge and satisfaction in an undergraduate course. Therefore, ideally, we propose the use of GBL in general, particularly Kahoot! in face-to-face classes to increase students’ satisfaction with the course [26]. The impact of the COVID-19 pandemic on medical education prompted a rapid shift to online teaching for higher education students, such as medical students, which has been partially filled with the GBL system [18, 27]. Regardless of the circumstances conditioning the teaching methodology, the pedagogical end goal of any GBL system is to maximize student learning by increasing students’ participation, attentiveness, motivation and satisfaction and decreasing students’ anxiety about learning the subject [28–31].

Studies have demonstrated that GBL, including the Kahoot! methodology, is a valid tool that can be used as an alternative to the traditional learning methodology. In this regard, we would like to highlight the study of Figuccio et al. [32], which revealed that review classes given to 190 students in the education degree program led to the same results on the final exam regardless of the methodology used (the Kahoot methodology vs. the traditional methodology). Likewise, Gözde et al. reported that intramuscular injection knowledge was acquired significantly better by 110 nursing students using the Kahoot! methodology than through face-to-face education [33]. In addition, studies have shown that Kahoot! could be an optional tool (vs. paper) to quickly summarize the essential content in medical school class lectures [32, 34–36]. One of the most valuable features of a game such as Kahoot! that is positively appreciated by students is that it allows students and the instructor to receive immediate feedback on their work and progress on their knowledge acquisition. Most of the data in the literature evaluating the use of Kahoot! rely on students’ self-reports and qualitative assessments. However, to our knowledge, no studies have examined the direct impact of Kahoot! from a quantitative perspective. Our study shows that there is a positive and significant correlation between Kahoot! exercises and exam grades. In addition, we found that students who completed Kahoot! exercises had significantly higher values for each exam.

Studies assessing the use of the Kahoot! methodology to improve final grades have shown contradictory results. For instance, Harrelson et al. and Yuenyongviwat and Bvonpanttarananonand et al. [34, 35] reported that students in classes using Kahoot! did not have significantly better scores on their final examinations than students in a control group. However, it is important to highlight that these studies did not include the use of Kahoot! as an additional reinforcement tool but as an alternative system to a traditional quick paper. In contrast, our study demonstrated that grades were significantly higher in the academic year when Kahoot! was used than in the “traditional” year for the human histology subject. This may be because the performance on Kahoot! was conducted in seminars to reinforce and review each subject topic. These results seem to be in agreement with those obtained by Bawa et al. [37] with business course students, who reported that the use of Kahoot! improved their final grades. Thus, we conclude that Kahoot! used throughout an academic course is a powerful tool that can be used to reinforce the knowledge acquired and improve students’ final grades.

In addition to its ability to improve final grades, we wanted to explore the possible predictive value of the tool. To achieve this, Kahoot! was implemented in another subject that has not previously been exposed to GBL methodologies. The students had only one Kahoot! attempt prior to the final exam to avoid becoming familiar with the nature of the questions for the final exam. Our results showed a significant correlation: students with better results in Kahoot! obtained better final grades, and those with worse results in Kahoot! obtained worse final grades. Consequently, we concluded that Kahoot! is a potential predictive tool for final grades. Kahoot! is also an efficient tool for evaluators to predict students’ acquired knowledge, allowing evaluators to identify those with the greatest difficulties as well as the contents or concepts that must be reviewed.

Our study has some limitations that should be noted. In both subjects, the Kahoot! exercise was performed in class. Since the classes were not compulsory, it was only completed by the students who normally attended the classes. One of the limitations that should be taken into consideration when interpreting our results in the human histology subject is the comparison of different courses composed of different students with distinctive personalities and backgrounds. Therefore, the results obtained in the comparisons must be interpreted with caution since we do not know whether there were previous differences between the two groups. However, medical students are a very homogeneous academic population because discharge requires a cohort grade for admission. This high mark for admission generates a uniform population in terms of academic capacities. The year 2019–2020 was not included to prevent any effects of the COVID period on class attendance and learning effectiveness that might affect the results. With regard to the implementation of the tool, when Kahoot! is used several times throughout the course, students might become familiar with the nature of the questions and important concepts of each topic that are likely to be assessed in the exam.

In the case of neuroanatomy, there was no control group. Having half of the group perform Kahoot! online would be unfair and would contradict the overall teaching principles. Finally, random responses from students who did not know the answers should have been taken into account, including item response theory (IRT) in the Kahoot! exercise [38]. However, random responses were analyzed as outliers in Kahoot responses [39].

## Conclusion

According to our results, students who completed the Kahoot! exercise had significantly higher grades than students who did not complete it. Therefore, our study shows for the first time that Kahoot! can be used as a method of forecasting the final grade and is useful for both teachers and students. We conclude that the benefits of using Kahoot depend on the method of implementation of the tool. When used periodically during the course, it reinforces knowledge acquisition and improves final grades, whereas when used as a unique session prior to the exam, it has potential predictive value for the final grade.

## Electronic supplementary material

Below is the link to the electronic supplementary material.

Supplementary Material 1

## Data Availability

The datasets used and/or analyzed during the current study are available from the corresponding author on reasonable request.
