# Supplementary material for: Performance in Kahoot! activities as predictive of exam performance
Source: BMC Med Educ. 2023 Jun 6;23:413. doi: 10.1186/s12909-023-04379-x (PMC10242591; doi:10.1186/s12909-023-04379-x)
Supplement: Supplementary file 1 — Supplementary Material 1 [file 12909_2023_4379_MOESM1_ESM.docx]

**Supplementary Table 1.** Description of the students included in the current study

| **Group** | **Total number of students enrolled** | **Sex distribution**  **(female/male)** | **Total number of students who did Kahoot exercise** | **Total number of students who did not make Kahoot exercise** |
| --- | --- | --- | --- | --- |
| Neuroanatomy group (2021-2022) | 173 | 127/46 | 125 | 48 |
| Histology group with traditional learning  (2018-2019) | 212 | 159/53 | 0 | 211 |
| Histology group with Kahoot exercise  (2020-2021) | 200 | 135/65 | 200 | 0 |

**Supplementary Table 2.**  Titles of theorical and practice class in the Neuroanatomy subject

| **Type of class** | **Title** |
| --- | --- |
| Theorical | Unit 1. Introduction to the Nervous System |
| Theorical | Unit 2.- Sense organs: Touch, taste and smell |
| Theorical | Unit 3.-Sense of Hearing: Inner ear, Middle ear, Outer ear |
| Theorical | Unit 4.- Sense of Sight. Eyeball, walls: Retina, Vascular layer, Fibrous layer |
| Theorical | Unit 5.- Sense of sight. Eyeball, content: Lens, Humores |
| Theorical | Unit 6.- Oculomotor neuromuscular systems |
| Theorical | Unit 7.- Orbital content and protection organs |
| Theorical | Unit 8.- Configuration, structure, vascularization and envelopes of the spinal cord |
| Theorical | Unit 9.- Medullary sensory systems |
| Theorical | Unit 10.- Vegetative effectors in the medulla |
| Theorical | Unit 11.- Somatic motor effectors in the medulla. Spinal cord reflexes |
| Theorical | Unit 12.- Configuration, structure and vascularization of the brainstem |
| Theorical | Unit 13.- Brainstem sensory systems |
| Theorical | Unit 14.- Vegetative effectors in the brainstem |
| Theorical | Unit 15.- Somatic motor effectors in the brainstem |
| Theorical | Unit 16.- Cranial nerves |
| Theorical | Unit 17.- Reticular formation |
| Theorical | Unit 18.- Colliculi and Substantia Nigra |
| Theorical | Unit 19.- Brainstem nuclei related to the cerebellum |
| Theorical | Unit 20.- Configuration, vascularization and envelopes of the cerebellum |
| Theorical | Unit 21.- Structure of the cerebellum |
| Theorical | Unit 22.- Archicerebellum/Vestibulo-Cerebellum |
| Theorical | Unit 23.- Paleocerebellum/Spino-Cerebellum, Neocerebellum/Cerebro-Cerebellum |
| Theorical | Unit 24. - Configuration, structure and vascularization of the diencephalon. circumventricular organs |
| Theorical | Unit 25.- Hypothalamus. Hypophysis, |
| Theorical | Unit 26.- Epithalamus. Pineal gland |
| Theorical | Unit 27.- Thalamus |
| Theorical | Unit 28.- Subthalamus. Telencephalic basal nuclei |
| Theorical | Unit 29.- Internal configuration of the telencephalon. white matter |
| Theorical | Unit 30.- Internal configuration of the telencephalon. Lateral ventricles. |
| Theorical | Unit 31.- External configuration of the cerebral cortex. |
| Theorical | Unit 32.- Structure of the cerebral cortex |
| Theorical | Unit 33.- Sensory cortices: somatoesthetic, acoustic and visual |
| Theorical | Unit 34.- Gustatory and olfactory cortex |
| Theorical | Unit 35.- Hippocampus and limbic system |
| Theorical | Unit 36.- Association cortices and frontal lobe |
| Theorical | Unit 37.- Motor cortices |
| Theorical | Unit 38.- Arterial vascularization of the brain |
| Theorical | Unit 39.- Venous return of the brain |
| Theorical | Unit 40.- Meninges and CSF circulation. brain barriers |
| Theorical | Unit 41.- Sensory pathways |
| Theorical | Unit 42.- Somato-motor pathways |
| Theorical | Unit 43.- Viscero-motor pathways |
| Theorical | Unit 44.- Radiological anatomy and image study techniques of the Nervous System |
| Practical lesson | Study of the oral and nasal cavities: taste and smell |
| Practical lesson | Study of the organs of hearing and balance. Boulder |
| Practical lesson | Study of the organs of vision. orbital cavity |
| Practical lesson | Macroscopic study of the marrow |
| Practical lesson | Study of medullary sections |
| Practical lesson | Macroscopic study of the brainstem |
| Practical lesson | Study of brainstem cuts and reconstructions |
| Practical lesson | Macroscopic study of the cerebellum |
| Practical lesson | Study of cuts and reconstructions of the cerebellum |
| Practical lesson | Macroscopic study of the diencephalon |
| Practical lesson | Study of cuts and reconstructions of the diencephalon |
| Practical lesson | External and internal macroscopic study of the telencephalon |
| Practical lesson | Study of sections and reconstructions of the telencephalon |
| Practical lesson | Study with imaging techniques of the Nervous System |

**Supplementary Table 3.** Questions included in Kahoot exam in Neuroanatomy subject.

| **Questions** | **Answer options** |
| --- | --- |
| **Question 1:** Which of the following structures passes through the sphenoid notch outside of the Zinc tendon? | a. Pathetic nerve  b. Nasal nerve  c. Ophthalmic artery  d. Superior ophthalmic vein |
| **Question 2:** What is the origin of the parasympathetic motor innervation of the parotid gland? | a. Dorsal nucleus of the vagus  b. Superior salivary nucleus  c. Inferior salivary nucleus  d. Lacrimuconasal nucleus |
| **Question 3:**  Why do you ascend the body's deep discriminative and kinesthetic information? | a. Ventral spinocerebellar tract  b. Bulbotalamic bundle  c. Anterior spinothalamic tract  d. Espinoolivar tract |
| **Question 4:**  What is the nature of the hypoglossal nucleus? | a. Viscero-motor  b. Somatic-motor  c. Sensible  d. Viscero-vegetative |
| **Question 5:**  Which nerve collects taste information from the posterior third of the tongue? | a. Vagus nerve  b. trigeminal nerve  c. Facial nerve  d. Glossopharyngeal nerve |
| **Question 6:** Which artery supplies the anterior spinothalamic tract? | a. Posterior spinal  b. Root artery  c. Anterior spinal  d. Commissural spinal |
| **Question 7:**  Which nerve will exit between the superior cerebellar artery and the posterior cerebral artery? | a. Pathetic nerve  b. Common oculomotor nerve  c. Trigeminal nerve  d. External oculomotor nerve |
| **Question 8:**  What is the name of the decussation of the fibers that leave the tectal nuclei to the spinal cord? | a. Forel's decussation  b. Meynert's decussation  c. Werneking's decussation  d. Pyramidal decussation |
| **Question 9:**  What type of information reaches the main trigeminal nucleus? | a. Epicritic somatosensory  b. Protopathic somatosensory  c. Gustatory somatovegetative  d. Cardiorespiratory somatovegetative |
| **Question 10:**  What is the structure that covers the superior surface of the cerebellum called? | a. Falx cerebellum  b. Sickle brain  c. Tentorium cerebelli  d. Pia mater |

**Supplementary Table 4.** Questions included in Kahoot exam in Histology subject.

| **Questions** | **Answer options** |
| --- | --- |
| 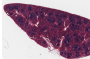**Question 1:** ¿Which is this organ? | a. Encapsulated lymphoid tissue: lymphoid organ  b. Mucosal associated secondary lymphoid follicles  c. Primary lymphoid organ  d. Cells of the immune system that are not considered as lymphoid tissue |
| **Question 2:** ¿What does observe in this image?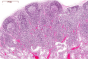 | a. Lymph node  b. Non-encapsulated lymphoid tissue  c. Mucosa-associated lymphoid tissue  d. White pulp of the spleen |
| **Question 3:**  ¿Which is this organ/tissue?  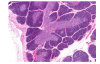 | a. Spleen  b. Secondary lymphoid organ: lymph node  c. Thymus  d. Mucosa-associated lymphoid tissue: pharyngeal tonsil |
| **Question 4:**  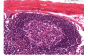This image shows: | a. Periarterial lymphoid sheath  b. White pulp of spleen  c. Primary lymphoid follicle of lymph node  d. None is correct |
| **Question 5:**  This image shows:  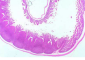 | a. Mesenteric lymph node  b. Peyer's plaque  c. Bronchus-associated lymphoid tissue  d. None is correct |
| **Question 6:** The arrow points to:  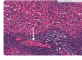 | a. Sinusoid  b. Trabecula  c. Central artery  d. None of these are entirely true |
| **Question 7:**  The arrow points to:  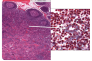 | 1. Lymphatic vessel 2. Muscular venule 3. Lymphatic sinus 4. High endothelial venule |
| **Question 8:**  Which histological structures you can see in the image?  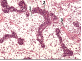 | 1. Splenic red pulp 2. Lymph node medulla 3. Splenic medulla 4. Degenerating thymus |
| **Question 9:**  Which one is correct?  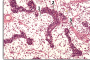 | 1. Sinusoids 2. extravasated blood cells 3. Splenic or Billroth's cords 4. Trabeculae in medulla of lymph node |
| **Question 10:**  In this image we observe:  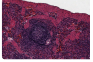 | 1. Trabeculae in the cortex superfitial of the ganglion 2. Splenic trabeculae 3. Marginal lymphatic sinuses 4. Exclusively splenic white pulp |
| **Question 11:** In this image we observe:  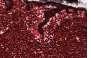 | a. Reticular fibers in marginal and cortical sinuses  b. red pulp sinusoids  c. Elastic fibers in capsule of the spleen  d. None is correct |
| **Question 12:** In this image we observe:  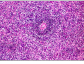 | a. periarteriolar lymphoid sheath  b. central artery  c. Diffuse lymphoid tissue replete with T lymphocytes  d. all are true |
| **Question 13:** This image correspond to:  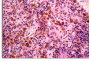 | a. Spleen  b. involuted thymus  c. Bone marrow  d. lymph node marrow |
| **Question 14:** In this structure, the cell that has the function of blood filtering is:  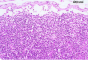 | a. macrophage  b. antigen presenting cells  c. Fibroblastic reticular cells  d. There are no cells with this function in the image |
| **Question 15:** The circle is pointing to:  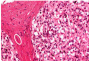 | a. reticuloepithelial cell "cytoreticulum"  b. collagen fiber  c. smooth muscle fiber  d. reticular fiber |
| **Question 16:** In this image we observe:  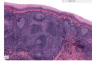 | a. No diffuse lymphoid tissue  b. Dense connective tissue capsule  c. Encapsulated lymphoid tissue  d. Mucosa-associated lymphoid tissue |
| **Question 17:**  What cells conform these characteristic structures?  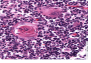 | a. Type I broblastic cells  b. Type IV interdigitated dendritic cells  c. Reticuloepithelial cells type 2  d. Type V reticular epithelial cells |
| **Question 18:**  The area marked by the asterisk is:  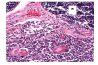 | a. thymic medulla  b. white pulp  c. thymic cortex  d. Lymph node cortex |
| **Question 19:**  The lights correspond to empty splenic sinusoids.  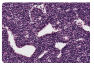 | a. True  b. False |
| **Question 20:**  The number 4 marks:  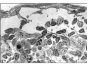 | a. The reticular fibroblastic cells of the lymphatic sinuses  b. Macrophages  c. Lymph cells  d. Reticular fibroblasts in splenic sinusoids |
| **Question 21:**  The arrow indicates  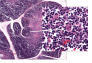 | a. Macrophages engulfing apoptotic lymphocytes  b. Hassal's Corpuscle  c. Type 3 reticuloepithelial cells at the medullary corticolimitation  d. I don't know, it can't be determined without doing immunohistochemistry |
| **Question 22:**  Which one is correct?  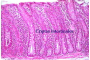 | a. Isolated lymphoid cells  b. You can NOT talk about lymphoid tissue  c. Diffuse lymphoid tissue  d. Lymphoid follicles |
| 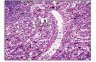**Question 23:**  The number 1 is pointing to wide capillaries draining into trabecular veins | a. True  b. False |
| **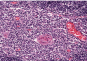Question 24:**  This micrograph shows the splenic white pulp | a. True  b. False |
| **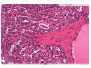 Question 25:** These nuclei belong to: | a. The endothelium of splenic sinusoids b. The wall of lymph sinuses  c. Limphocytes  d. High endothelial venules |
